# Applying Change Models and Methods During a Period of Vast Digital Transformation: A Systematic Review of Practice in Healthcare

**DOI:** 10.1002/hpm.70092

**Published:** 2026-05-21

**Authors:** Rebecca McDonnell, Ashfaq Chauhan, Corey Adams, Alexander Cardenas, Michelle Moscova, Ramya Walsan, Maryam Sina, Alice Munro, Elizabeth Manias, Rebecca Mitchell, Anthony Gust, Sabe Sabesan, Reema Harrison

**Affiliations:** ^1^ Trainee Educational Psychologist University College Dublin Dublin Ireland; ^2^ Centre for Health Systems and Safety Research Australian Institute of Health Innovation Macquarie University Sydney New South Wales Australia; ^3^ Health Infrastructure NSW St Leonards New South Wales Australia; ^4^ Faculty of Medicine and Health UNSW Sydney Kensington NSW Sydney Australia; ^5^ Western NSW Local Health District NSW Australia Majarlin Kimberley Centre for Remote Health Notre Dame Australia; ^6^ School of Nursing and Midwifery Monash University Victoria Australia; ^7^ Centre for Healthcare Resilience and Implementation Science Australian Institute of Health Innovation Macquarie University Sydney New South Wales Australia; ^8^ Northern Health Victoria Australia; ^9^ Townsville Cancer Centre Townsville University Hospital Townsville Australia

**Keywords:** change management, digital health, health systems, healthcare leadership, virtual care integration

## Abstract

**Introduction:**

Reflection and learning about the use of virtual care in healthcare delivery has become a central goal for health systems internationally. Insights drawn in the aftermath of the COVID‐19 pandemic have led to vast changes to embed virtual care in health care delivery. This study explored the methodologies used to manage change that encompasses virtual care and factors contributing to success.

**Methods:**

A systematic review and narrative synthesis was undertaken. Eligible articles were those reporting structured change management processes in the context of virtual care published between 1st January 2019–31st December 2023, identified by searching four electronic databases (Scopus, MedLine, PsycInfo and Business Source Premier). Data were extracted and synthesised from the eligible studies.

**Results:**

Seventeen studies met inclusion criteria describing changes occurring within hospital settings or in community health centres. Kotter's 8‐Step Model was the most frequently applied change framework, often combined with other approaches. Commonly enablers included high quality communication among all parties involved and strong leadership. Common barriers included overemphasis on technology at the expense of people and processes, linear application of models, and lack of mechanisms to monitor change progress.

**Discussion:**

Structured change methodologies were often integrated in a strategic change framework with process improvement methods utilised to support the change process. Managing change relating to the technology with attention to the clinical and people aspects of change was considered a key gap and challenge in the context of virtual care change. Change leadership and the integration of technical and clinical teams were identified as key enablers.

## Highlights

- Structured change management models have been adopted to integrate virtual care
- The role of change management in the success of virtual care change is limited
- Technology, people and process change must be considered together
- Incremental introduction of technology may improve virtual care change success

## 1 Introduction

Virtual care, includes care that is delivered via telephone, video‐conferencing and remote‐monitoring of health conditions. Delivery of virtual care is supported by digital health tools and technologies such as digital patient information systems, electronic medical records (EMRs) and other digital infrastructure [1, 2, 3]. The COVID‐19 pandemic triggered an unprecedented and rapid shift toward virtual and hybrid (combining virtual and in‐person services) models of care. This transformation represents one of the most substantial periods of change experienced in healthcare in recent history; both in the volume of services and individuals impacted, and the types of changes required worldwide [4, 5]. For example, in March 2020, the Australian Government expanded Medicare Benefits Schedule (MBS) telehealth items, enabling phone and video consultations for all Australians, regardless of location. This required health services to rapidly adapt to this change and integrate virtual models of care [4]. Integration of virtual models of care is characterised by digital transformation or introduction of new technologies, system ownership and monitoring, and coupled with organisational change impacting teams and workflows [6, 7, 8]. Successful integration and ongoing use of virtual and hybrid models of care provision are contingent upon transition approaches to information sharing. These include aligning organisational culture, management and approaches to effective teamworking in virtual and hybrid contexts, and appropriately upskilling the workforce to leverage technologies, identify risks and engage in virtual and hybrid working effectively [9, 10].

The digital tools and technologies that are introduced require changes to established ways of working, which can have an impact on organisational culture, structure, existing workflows, and interpersonal dynamics [7, 11]. Establishing trust, inclusive teams, effective teamwork approaches, and the technical and employee support systems within organisations, along with methods to monitor the process and outcomes of change are identified as important [12, 13]. In healthcare settings, both staff and consumers are faced with the dual adjustment of learning how to engage with new tools and technologies alongside the wider effects that these tools and technologies may have on the process of delivering and receiving healthcare.

Unparallelled workplace changes necessitated in the past 5 years have enabled organisations worldwide to determine the work that can be undertaken effectively at distance, and the circumstances in which on‐site or in‐person interaction is advantageous or required. A body of literature has also examined the creation of new work conditions as a result of digitisation, and the role of change management in these processes [14, 15]. International case studies of digital transformation in healthcare organisations identify factors for successful digitisation, highlighting achieving change and digital transformation readiness among staff and recognising digitisation as greater than technical change as critical factors [16, 17, 18, 19]. Despite featuring a range of change management activities, these case studies omit discussion of a planned approach to managing the change associated with digitisation [20, 21].

Despite widespread recognition of the scale of change associated with the rapid digitisation of care, there has been limited exploration of how change management models and methods have been applied in healthcare organisational management approaches. A review (published in 2021) examining application of change methodologies in healthcare identified 31 studies; however, application of change models and methods to integrate virtual care was not explored [22]. Increased use of virtual models of care presents new possibilities for improving healthcare efficiency and experiences, whilst reducing disparities in healthcare access [23, 24]. Learning from integration of virtual care is important to further capitalise on the opportunities for service improvement identified through the pandemic period. This provides an opportunity to consider what has been learnt about the management of change in the context of shifting to integrating virtual care in healthcare delivery. This systematic review therefore aimed to identify and synthesise contemporary evidence of the use of change management models and methods in the context of changes that relate to the use of virtual care in healthcare delivery. The review questions were: (1) What are the change management models and methods that have been used in the context of changes relating to use of virtual care? (2) How have these change methodologies been applied and to what effect? and (3) What factors are barriers or enablers to successful changes relating to use of virtual care?

### 1.1 Ethics Statement

Not applicable as this was a systematic review.

## 2 Methods

### 2.1 Design

A systematic literature review was conducted and reported in accordance with the Preferred Reporting Items for Systematic Reviews (PRISMA) [25].

### 2.2 Eligibility Criteria

– *Inclusion Criteria:* Articles were eligible for inclusion that:
  - Reported a process of change within any healthcare service or system setting in which a defined change management model or method was used. For this review, change management models or methods were defined as ‘structured overall processes for change from inception of change to benefits realisation’ [22];
  - Were published between 1st January 2019–31st December 2023 to focus on the pandemic period and beyond whereby widescale and rapid change to use of virtual care occurred;
  - Focused on change in relation to the incorporation of, or transition to, virtual care in health services, which was defined broadly *as the use of any digital tools to enable the delivery of any aspect of healthcare provision*; and
  - Used any study design.
– *Exclusion Criteria:* Articles that were non‐primary sources, not published in English, published prior to 1St January 2019, discussed or recommended hypothetical changes that were not implemented, or that did not specify a change management model or method were excluded. Literature reviews, systematic reviews, non‐peer reviewed and discussion pieces were excluded; however, their reference lists were reviewed for relative empirical studies. Conference and poster abstracts, opinion pieces, or letters were also excluded. Studies that conducted implementation evaluation of virtual models of care using implementation science frameworks were also excluded as they did not identify change management models or methods.

### 2.3 Study Identification

A search strategy (Supporting Information S1) was developed in consultation with a university librarian with expertise in healthcare. The strategy used keywords and synonyms focused on the concepts of change management, healthcare delivery, digitisation and virtual healthcare. The search strategy was applied to four databases: Scopus, MedLine, PsycInfo and Business Source Premier in June 2023 and a further search update was conducted in February 2024.

### 2.4 Study Selection and Data Extraction

Three reviewers (R.M., C.A., A.C.) independently screened the titles and abstracts of the selected studies against the eligibility criteria. Papers that met the inclusion criteria based on title and abstract were subject to a full text review. A fourth reviewer conducted a final check on the articles selected for inclusion against the eligibility criteria (R.H.). The following data were extracted from the included studies; lead author, year published, study design, country, timeframe, setting, sample size, change management model or methods, study objective, key findings, enablers and barriers to change management.

### 2.5 Data Synthesis

Narrative synthesis [26] was conducted using a team‐based approach with the authorship team. Following the tabulation of initial descriptions of the included studies, the change management approaches and methods, and the resulting impact on the management of change, team members individually reviewed the included articles. The group then met to discuss key findings, explore commonalities in current approaches and techniques that had been successfully applied, and identify the challenges and mitigation strategies. Through this group discussion, initial themes were generated to describe the available evidence. Four research team members developed the results content and shared this with the wider group for further development. Interrogation of the findings considered publication characteristics and their results; the models and methods used; and the influence of context on the application of change management approaches.

### 2.6 Assessment of Study Quality

As the included studies adopted a case study approach, methodological and reporting quality appraisal was conducted using the Critical Appraisal Checklist for a Case Study developed by the Centre for Evidence Based Management (CEBMa) [27]. This tool comprised 10 items and for each item, the response was recorded as ‘Yes’, ‘No’ or ‘Can't tell’.

## 3 Results

### 3.1 Results of the Search

**FIGURE 1:**
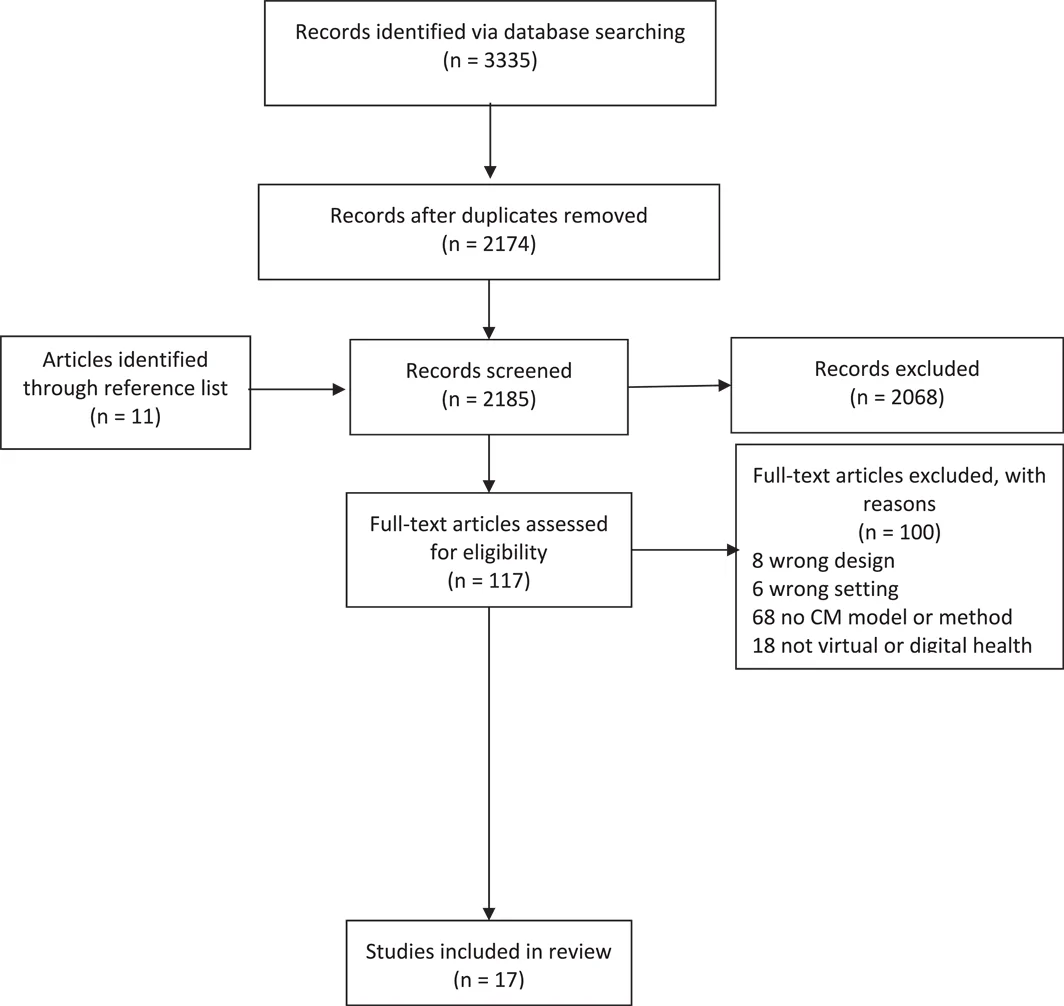
PRISMA flow diagram describing the selection of studies included in the review.

A total of 3335 articles were extracted from EndNote X20 into Covidence. Once duplicates were removed and title and abstract screening was conducted, 117 papers were eligible for full text screening. Seventeen studies met the eligibility criteria for this review. Figure 1 outlines the screening and selection process.

### 3.2 Excluded Studies

As shown in Figure 1, 100 studies were excluded after full‐text review. The main reasons for exclusion included failure to discuss a structured change management process or method (*n* = 68 studies), there was no virtual care component to the change (*n* = 18 studies), studies that were not peer‐reviewed (*n* = 8 studies) or study setting other than the healthcare system setting (*n* = 6 studies).

### 3.3 Characteristics of Included Studies

A total of 17 studies were included in the review (Table 1); these originated from the United States of America (US; three studies) [28, 29], the United Kingdom (UK; three studies) [30, 31, 32], Australia (three studies) [21, 33, 34], Canada (two studies) [35, 36], Saudi Arabia (two studies) [37, 38], Brazil (one study) [39] and Taiwan (one study) [40]. One study was conducted across the European Union [41] and one manuscript was a synthesis of digital change case studies conducted across different countries [42]. Included articles were all case studies and these case studies predominantly focused on changes occurring within hospital settings (15 studies) but also in community health centres (two studies) [28, 34]. Most studies reported changes occurring within a single organisational unit (such as ward or unit) (five studies) [33, 34, 39, 40, 43], a single hospital (four studies) [21, 35, 37, 38] or across hospital networks (e.g., as National Health Service Trust) (five studies) [28, 30, 31, 32, 36], healthcare systems (one study) [29] and across multiple countries (two studies) [41, 42] were also identified.

**TABLE 1:** Study characteristics.

Authors and year	Setting	Project, study design and method	Sample	Objectives	Virtual or digital change initiative undertaken	Structured change method/process used	Key conclusions
Arabi et al. 2022 [37]	Tertiary care hospital, Kingdom of Saudi Arabia	Case study. Narrative reflection with quantitative measures.	Various examples are provided with range of sample size based on the indicators used.	To describes the leadership aspects of a large‐scale EMR implementation using Kotter's change management model.	The new EMR system included patient medical record, computerised physician order entry (CPOE), clinical decision support system (CDSS), closed‐loop medication administration (CLMA), clinical data warehouse (CDW), health information exchange (HIE), and disaster recovery (DR).	Kotter's 8 step change model.	Project conclusions The EMR was associated with: – High rating of patient and staff satisfaction – Reduction in waiting time for the appointment. – Improvement in management of glycaemic control Change process conclusions: Specific set of leadership competencies are required to implement complex adaptive IT based EMR implementation. Kotter's change model allowed for successful integration of IT model.
Baslyman et al. 2019 [38]	Tertiary care hospital, kingdom of Saudi Arabia	Case study. Discuss three case scenarios.	NA	A case study example of assessing and implementing RTTS system in one hospital.	Implementation of real‐time tacking sample (RTTS) system meant to track samples in real time and report on the current position of samples while travelling from the ED to the lab.	Lean change management—Lean AbPI (activity‐based process integration) model.	Project conclusions: The Lean‐AbPI model was also more cost effective than prior model that was used to manage lab sample deliveries. Change process conclusions: The Lean‐AbPI model is proposed as a new model that uses the requirements engineering (RE)‐based analysis methods within change management approaches. The Lean‐AbPI model supported leaders in reaching more concrete conclusions and providing better traceability to decisions.
Bird 2020 [30]	One NHS trust, UK. Five inpatient wards	Case study.	NA	To describe the methods of change management used to roll out the new workflows involved in the electronic barcode medicines administration process and to ensure they were fully adopted by staff in the clinical areas.	Implementation of a barcode medicines administration system.	Kotter's 8 step change and Lewin's three step models.	Project conclusions: Compliance rate in one a pilot ward was sustained even after non‐clinical IT staff that supported change in this ward were moved to assist with change in two different wards. Change process conclusions: The adoption rates of the new workflow patterns have shown the benefits of using different change management models during various phases of a project. No single model of change management is perfect and change agents must use a wide range of resources and models to successfully implement a new project.
Bousquet et al. 2019 [41]	Allergic rhinitis management across European Union	Case study.	NA	To increase self‐medication and shared decision making in rhinitis and asthma multimorbidity through development and validation of information technology evidence based tools.	Development and validation of mobile airways sentinel network (MASK) as an information technology evidence‐based tool. The mobile technology of MASK is the allergy diary available as a fee app in 23 countries in 16 languages.	A new maturity CM model based on Kotter's 8 step model. The article discuss the Kotter's model with lewin three‐step and Lippitt's six‐step planned change management models.	Project conclusions: No results were discussed in detail other than a point noting that patients with allergic rhinitis (and possibly with asthma) do not follow physicians' advice: They self‐medicate. Change process conclusions: The manuscript proposes a change management tool based on Kotter's change model to implement MASK ARIA tool as an allergy diary.
Burns 2023 [31]	One NHS trust, UK. Across the whole trust.	Case study. NA	NA	To describe the process of electronic patient record (EPR) integration and its impact upon manchester university national health service foundation trust health information management teams.	Implementation of integrated EPR system across a large NHS trust.	A structured change process that consisted of pre‐implementation preparatory work, deliverables in form of staff training, data quality programme and clinical coding, implementation of change if form of go‐live and evaluation post go‐live was undertaken.	Project conclusions: A single go live at the largest NHS trust in England was delivered. Benefits in various areas have been realised. For example, patients have improved access to their data and improvements noted in operating theatre utilisation. Change process conclusions: Success attributed to organisation wide commitment and executive support. Clinician lead process with clinicians trained as champions and provided support and training to lead the change.
Carman et al. 2019 [28]	Eight federally run community care clinics, US	Case study. Qualitative description.	12 clinic staff.	To understand how a federally qualified health centre (FQHC) in rural Kentucky implemented a significant organizational change to improve its delivery of preventive care services, close care gaps, and reduce health disparities.	Integration of proactive office encounter (POE)—An electronic tool to identify and track chronic care patients.	Kotter's 8 step change model.	Project conclusions: No direct project conclusions outlined. Change process conclusions: Inclusion of all of Kotter's steps ensures that appropriate leaders in the organization guide such a change, that personnel involved in the change understand its purpose, and that the project is managed to the point of anchoring the change in the organization's culture. Step 8—Anchoring the change in organisational culture was considered as an area for improvement.
Chang et al. 2019 [40]	Inpatient oncology unit, taiwan	Quantitative.	NA	To reduce falls by implementing a fall reduction plan including the “traffic light” fall risk assessment tool (TL‐FRAT).	Implementation and use of new traffic light—Falls risk assessment tool in the electronic medical record system.	Programme evaluation design grounded in Kotter's 8 step change model.	Project conclusions: The average fall incidence and falls with injury during the project were reduced. Change process conclusions: No change process conclusions provided.
Dyudina 2022 [42]	Not applicable	Synthesis of cases from multiple countries	NA	To present a comparative analysis of the stages of synchronisation of the digital transformation or medical institutions.	Analysis of digital transformation of medical institutions.	Lean methodology as an overarching application tool to the cases	Project conclusions: Not applicable Change process conclusions: The author presents a case for digital transformation case to be carried out not within the framework of individual elements of digitisation of activities of a medical institution but using an integrated approach based on lean production system.
Eden et al. 2022 [21]	Tertiary hospital, Australia	Case study. Qualitative data from interviews and observations and publicly available project records before and after implementation.	NA	To describe the process of digital transformation of one hospital.	Implementation of integrated electronic medical record systems consisting of electronic medical record, computerised provider order entry, ePrescribing and computerised decision support.	Four stages of digital transformation in one hospital—creating a vision, clinical involvement, supporting initial use and transition to ongoing use.	Project conclusions: Not applicable Change process conclusions: The reflective articles provide a case example of a successful implementation of ieMR system at one hospital and describes steps for implementation across the wider health system. Need for executive role in balancing tension across different sites is outlined.
Glegg et al. [35]	Paediatric health service, Canada.	Case study.	NA	To describes the development and implementation of a custom‐designed excel‐based visual resource management tool	Implementation and use of excel visual resource management tool.	ADKAR change model and LEADS leadership framework	Project conclusions: Team members reported high perceived effectiveness and efficiency with respect to the tool's utility in supporting its proposed aims. Change process conclusions: Development of the excel tool and implementation using established leadership frameworks ensured collaboration, utility and acceptance of the change process. A graded approach to building knowledge and skills in using the tool, to individual responsibility for data entry, and to accountability by team members facilitated its successful implementation.
Krasuska et al. 2021 [32]	Secondary healthcare provider organisations, UK	36 case studies	Sample comprised of 628 interviews, 190 meetings observations and 499 documents analysis.	To qualitatively evaluate the impact of the global digital exemplar (GDE) programme in promoting digital transformation in provider organisations that took part in the programme.	Stimulating digital transformation across English NHS healthcare providers and to form a central point for facilitating knowledge creation by creating ‘global digital exemplars’ (GDEs)—local centres of digital excellence that could serve as examples of best practice.	The GDE programme—A cohort of provider organisations that would act as exemplars of digital excellence.	Project conclusions: GDE programme accelerated digital transformation within participating provider organisations. Change process conclusions: The change was achieved through protected funding, putting in place governance structures and through harnessing reputational benefits for participating provider organisations.
Lindsay et al. 2023 [29]	One academic healthcare system, US	Case study. Quantitative.	NA	To describe a documentation redesign to decrease nursing reassessment documentation time at an academic healthcare system using Kotter's CM theory.	Documentation process redesign within electronic health record.	Kotter's 8 step model	Project conclusions: The February‐April 2022 NEAT data reflected 129 min, a 20% decrease in documentation time. The time spent in flow sheets decreased from 58 min preintervention to 50 min, a 13% decrease. Change process conclusions: Kotter's change model provided an effective and practical framework to create sustainable change in nursing documentation workflow redesign. The model was simplistic and methodical for nurse leaders and engaged and empowered staff to contribute to the change vision. Key stakeholder selection was necessary to ensure leadership endorsement and staff participation. Motivating the team members led to engaged staff who incorporated the change as the new norm and standard.
Ma et al. 2023 [36]	Cardiac catheterisation facilities, state‐wide, Canada	Case study Quantitative surveys	NA	To describes the implementation of the contrast RISK decision‐support tool within Alberta, guided by the Canada health infoway change management model, and highlights relevant factors governing successful health technology implementation and evaluation.	Introduction of an electronic decision support tool with physician audit and feedback into all of the cardiac catheterisation facilities. Identified 6 key elements in the process of adopting electronic health tools in clinical practice: Governance and leadership, stakeholder engagement, communications, workflow analysis and integration, training and education, and monitoring and evaluation.	The Canada health infoway change management model	Project conclusions: Following implementation there were significant increases among health providers in the odds of: — Satisfaction with processes for identifying those at high risk of AKI. — Quantifying the appropriate level of contrast dye for each patient — Determining the optimal amount of IV fluid for each patient – Following up of kidney function of high‐risk patients Physicians uniformly agreed that the system was well‐integrated into existing workflows, while 42% of health providers also agreed. Change process conclusions: The Canada health infoway change management framework was a suitable model that underpinned the integration of new roles and responsibilities of health care providers.
Pizzini et al. 2019 [43]	Anaesthesia department within one medical centre, US	Case study.	NA	To provide a retrospective report on the affordances of how using Kotter's CM model during the pandemic supported with managing acute healthcare challenges.	Development and integration of an anaesthesia medication use dashboard using an excel template to analyse and compare its spending.	Kotter's 8 step model	Project conclusions: Anaesthesia medication dashboard use reduced the cost, from averaging $38,000 per month up to November 2016 on ephedrine alone to a little under $8000 for both ephedrine and epinephrine. Change process conclusions: The health service recognised that collaboration from a diverse group was required to understand the opportunity, outline alternatives, agree on the solution and create a new process.
Sale et al. 2019 [33]	Radiation oncology, one hospital, Australia.	Case study (redevelopment project) Method not specified.	NA	To describe the successful move of the radiation therapy department to its new site	Relocation of a technological unit.	Four step model of change (denial, resistance, exploration and commitment).	Project conclusions: The unit was successfully relocated. Change process conclusion: The four‐stage model of change assisted in the smooth implementation of a transition plan for radiation oncology unit. Staff coping mechanism need to be central in the change process. Well‐managed change involves people feeling supported through the process of change.
Tombocon et al. 2021 [34]	Renal dialysis centres, Two hospital networks, Australia	Case study (quality improvement project) quantitative.	NA	The primary aim was to develop and implement the new pathway and to improve home‐based dialysis therapy prevalence rates	Implementation of a service model with new technology and equipment.	Lean‐thinking framework, PDSA and Kotte's 8 step model.	Project conclusions: A target prevalence for home‐based therapies was achieved within 2 years. The prevalence rate reached 35% within 3 years and was maintained at 8 years. Change process conclusions: No change process related conclusions outlined.
Tortorella et al. 2022 [39]	Sterilisation unit, one teaching hospital, Brazil.	Case study. NA	10 staff	To propose value stream mapping technique that demonstrate how H4.0 digital applications can be combined with lean health to improve flow of value.	Implementation of digital healthcare with lean healthcare.	Based on lean and digital healthcare and DIT (diffusion of innovation theory) three step value stream model was used: 1. Map the current and future state. 2. Collect data on H4.0 digital applications and kaizen bursts. 3. Rank H4.0 digital application for value stream integration	Project conclusions: Value stream model demonstrated implementation of H4.0 digital applications in healthcare value streams. Change process conclusions: The systematic and concurrent implementation of LH and H4.0 supported by the proposed method allows mitigating scattered and shallow improvement initiatives.

A variety of changes were reported relating to the use of virtual care or digital tools, including managing change during the introduction of large digital systems such as EMR systems or medicine barcoding systems (seven studies) [21, 30, 31, 32, 37, 38, 42], embedding new digital tools to assess or track patients (six studies) [28, 35, 36, 40, 41, 43], the introduction of new technologies or digitally enabled equipment into care (two studies) [33, 34], electronic documentation redesign to improve efficiency (one study) [29] or process redesign using digitalised documentation (one study) [39].

### 3.4 Study Quality

Study quality varied widely based on the CEBMa Critical Appraisal Checklist for Case Study scoring (Supporting Information S2). The included studies provided rich reports of the change management context and approach taken, often from within the organisation or service rather than from an external research lens. As such, the included studies described in rich detail the change processes and methodologies used, gaining higher quality appraisal scores in the reporting of research aims, use of study design appropriate to address these aims and drawing conclusions based on the research findings. Studies demonstrated variable strength across reporting items that assessed reporting of data collection activities, largely due to the inconsistencies in structure and reporting approach adopted in each case study report. As case studies, the articles were also limited in demonstrating the application of the findings to other settings.

### 3.5 Main Findings Against the Research Questions

#### 3.5.1 What Change Management Models and Methods Have Been Used in Changes Related to Virtual or Digitally Enabled Healthcare?

Kotter's 8 Step Model for managing change featured most predominantly (eight studies) [28, 29, 30, 34, 37, 40, 41, 43]. The eight sequential steps include creating a sense of urgency, building a guiding coalition, developing a vision and strategy, communicating the change vision, empowering employees for broad‐based action, generating short‐term wins, consolidating gains, producing more change, and anchoring new approaches in the culture. In the context of changes related to virtual or digitally enabled care, Kotter's model was used in projects ranging from large scale implementation of EMR [37] and medicines barcoding systems [30], through to changing nursing workflows [29] into programs that sought to reduce falls and improve patient self‐management [40]. Studies using Kotter's 8‐step model often described the model's application with reference to the process of strategic planning for the change, executive involvement, and engagement establishing executive and steering committees and communicating with staff about the need for the change [34, 43] with a focus on the desired future state. These studies reported the projects as having successful outcomes, but consideration of the specific contribution of the change approach used to achieve these outcomes was often omitted. Kotter's framework was however, described as easy to engage with for clinical staff and leaders who had limited experience of change management to allocate roles and responsibilities [28, 29].

Of the studies using Kotter's model, four combined the model with one or more other change management models, including Lewin's Three‐Step Change Model, Lippitt's Six‐Step planned change management model, programme evaluation design, and the Plan‐Do‐Study‐Act (PDSA) improvement science model [30, 34, 40, 41]. Lewin's model proposes a three‐step process of change: unfreezing (creating motivation for change), changing (implementing the change), and refreezing (reinforcing and stabilising the change). When applying Lewin's model together with Kotter, the included studies used Kotter as a framework for the overall process with Lewin's model embedded in this process, or each model for a different project stage. For example, Bird (2020) utilised Kotter's model for their pilot study on integrating new workflows involved in electronic barcode medicine administration. Thereafter Lewin's Change Management was used when scaling the project to the entire hospital [30]. While there was no discussion of the application of either model in the study, it was noted that Kotter's model was considered as better used for small scale change and Lewin's model for larger scale implementation [30].

Unlike the other methods applied, PDSA is a continuous improvement framework rather than a model and involves planning a change, implementing it, studying its effects, and then acting on what is learnt to make further improvements. Combining the Kotter and PDSA model was used in the context of developing and implementing a new pathway to improve home‐based dialysis therapy prevalence rates [34]. PDSA was used as a method to continually assess performance across the project cycle [34].

Two studies solely used Lean methodology [38, 42], in addition to two studies integrating Lean with other methods [34, 39]. Lean methodology is described as a way of organising human actions and behaviours to deliver more benefit and value by eliminating or reducing waste [34]. Lean was used only in case studies reporting specific examples of implementing a virtual or digital change. The remaining articles explored the application of a further seven models within the healthcare context: combining the ADKAR and LEADS Model (one study) [35], The Canada Health Infoway Change Management model (one study) [36], the Four Step Model of Change (one study) [33], and purpose‐built structured change approaches such as the Global Digital Exemplar (GDE) Programme (three studies) [21, 31, 32].

#### 3.5.2 How Have Change Methodologies Been Used and to What Effect?

All studies identified a structured process for managing change, but the extent to which the model or method was utilised in each project was not consistently reported, creating challenges in determining the extent to which change approaches were used or useful in achieving project aims. Success was defined variably in the studies; predominantly focusing on specific benefits sought by the projects in workflow, efficiency, and clinical processes rather than indicators of a successful process of change management.

##### 3.5.2.1 Local Level Change

Four studies reported local level changes within an organisational unit such as a clinic, department or ward [33, 39, 40, 43]. Kotter's model was used in two of the studies to make safety and efficiency improvements by reducing the incidence of falls on an in‐patient oncology unit [40], and reducing costs associated with anaesthesia medication use by developing and integrating a digital dashboard [43].

From the remaining two studies focusing on local level change, the 4‐stage change model, based on Kubler Ross' 5 stages of grief, was utilised to reflect on the transition of a radiology unit from one site to another in a large Australian hospital [33]. Given its retrospective methodology, the study highlights factors successful in using a staggered approach to introducing the new site by staff gradually migrating, which provided support in reassuring staff who were more apprehensive about the change when they later moved to the new site [33]. The second combined Lean Health and Diffusion of Innovation Theory into a three‐step Value Stream Model, which is applied early in change processes to determine where there is greatest value in transitioning to new digital tools [39].

##### 3.5.2.2 Institutional Change

Four articles reported activities conducted to facilitate institution‐level change [21, 35, 37, 38]. Two projects focused on EMR implementation [21, 37]; Arabi et al. (2022) applied Kotter's model to support the implementation of an EMR in a hospital in Saudi Arabia [37]. The study reported successful change in terms of EMR benefits realization, with a high rating of patient and staff satisfaction and had helped in improving processes of care and efficiency [37]. Patient waiting time from check‐in to the actual appointments averaged in hours in 2016, which was reduced to less than 4 minutes after EMR implementation in 2019 with appointment capacity reported as improved from an average of 2600 per day to 4000 per day. The authors acknowledged the project extended beyond a technological change and attributed improvements to more than simply EMR introduction with the integration of leadership capability in the management of change identified as central to success [37]. The importance of leadership capability to deliver effective change management was further recognized in Eden et al.’s (2022) EMR implementation [21]. Leadership was described as a central enabler in a third project seeking to introduce visual tools to enable programme planning and evaluation within a paediatric healthcare setting, drawing upon the ADKAR and LEADS frameworks [35].

A further six studies presented cases of multi‐institutional change across a series of health centres or hospitals [28, 29, 30, 31, 32, 36], three of which were based in the UK National Health Service (NHS). Using Kotter's model, but in conjuncture with Lewin's change model, Bird (2020) reported the transition into new workflows involved in using a new electronic barcode medicine system in an NHS Trust that comprised five UK hospitals [30]. Initially using Kotter's model for the local pilot work, and then using Lewin's model for the hospital‐wide rollout due to the greater perceived suitability of Lewin's model when applying change to larger cohorts because of its ability to acknowledge the need to ‘unfreeze’ from a current state when implementing change across a range of teams. The authors concluded that enhanced benefits may be achieved from using a variety of change models relevant to the different goals and stages of a change project [30]. A further NHS project in a Trust comprising of 12 hospitals employed a purpose‐built structured change approach to facilitate the introduction of an EMR system [31]. A central focus was on the migration of health record data with less focus on the process of wider change management; the authors concluded in the lessons learnt that performance management of the change process itself is important [31]. The third UK NHS project reported the use of a purpose‐built GDE Programme as a process to promote the transformative change in digital health occurring in leading UK NHS organisations and enable shared learning. The reputational benefits of being a recognised leader in digital transformation were identified as important in enabling organisational buy‐in for forthcoming projects and supporting a wider vision for change [32].

In the US, two studies applied Kotter's model across health centres [28, 29]. A case study by Carman et al. (2019) applied Kotter's model to understand how a federally qualified health centre (FQHC) in rural Kentucky implemented a significant and successful change to improve its delivery of preventive care services, close care gaps, and reduce health disparities among its patient population [28]. The effectiveness of the implementation was explored through interviews with healthcare staff by dividing Kotter's model into three tenets (Tenet 1: Steps 1–3, Tenet 2: Steps 4–6 and Tenet 3: Steps 7 & 8). The authors concluded that the model was valuable as guiding principles for planning and introducing the change, but that there was limited engagement with the model throughout the change implementation stages later in the project [28]. The second study confirmed the value of Kotter's model for providing a guiding structure for change leadership in a project that aimed to decrease nursing reassessment documentation [29].

A final multi‐institutional project in Canada sought to implement decision support tools into three health services using the Canada Health Infoway Change Management Framework that comprised of six components. Similarly to the findings of Burns et al., the benefits of engaging incumbent staff in the process of delivering the changes rather than introducing external staff to introduce change was noted. Health networks and services frequently relied on external providers to deliver training and manage key aspects of the change process, which created challenges for local engagement, buy‐in and sustainment of change [36].

##### 3.5.2.3 System Wide Change

Two system‐wide or multi‐country change projects were identified through this review [34, 41]. The largest study focused on change management in a World Health Organisation (WHO) project aiming to transfer Allergic Rhinitis and its Impact on Asthma (ARIA) from a general guideline to integrated care pathways using mobile technology [41]. Through employing a multi‐model approach integrating Kotter's, Lewin's and Lippitt's models across more than 70 countries worldwide, the article reports engaging with national allergy agencies and programmes yielding significant change in improving integrated care pathways for Allergic Rhinitis over an 18‐year period [41]. The use of change models provided a central approach to bring together a major and distributed change process, yet the authors highlight the top‐down nature of the models applied and the limitations of such approaches to address sustainment beyond a project lifecycle [41].

In studies that described the extent of application of the change model or method, studies with successful change projects were often characterised by models applied throughout the duration of the change process. For example, Tombocon et al. (2021) also utilised a combination of two models to change the vision and approach to dialysis care across two healthcare networks in an Australian state [34]. Kotter's 8 Step Model and the PDSA model formed the framework for developing a new pathway to improve home‐based dialysis therapy prevalence rates, with a vision for home‐based care before hospital care being central to the effort [34]. The study reported that using these change management processes at every point in the project aided in the implementation of a new model of care with a high patient uptake of home dialysis sustained over 8 years [34]. Engagement in the overall vision and its value enabled nurses at the forefront to lead on the change and patient engagement to be strong throughout, which were considered important factors contributing to the ability to sustain the new approach [34].

#### 3.5.3 What Factors Are Barriers or Enablers to Successful Changes Relating to Virtual or Digitally Enabled Healthcare?

**FIGURE 2:**
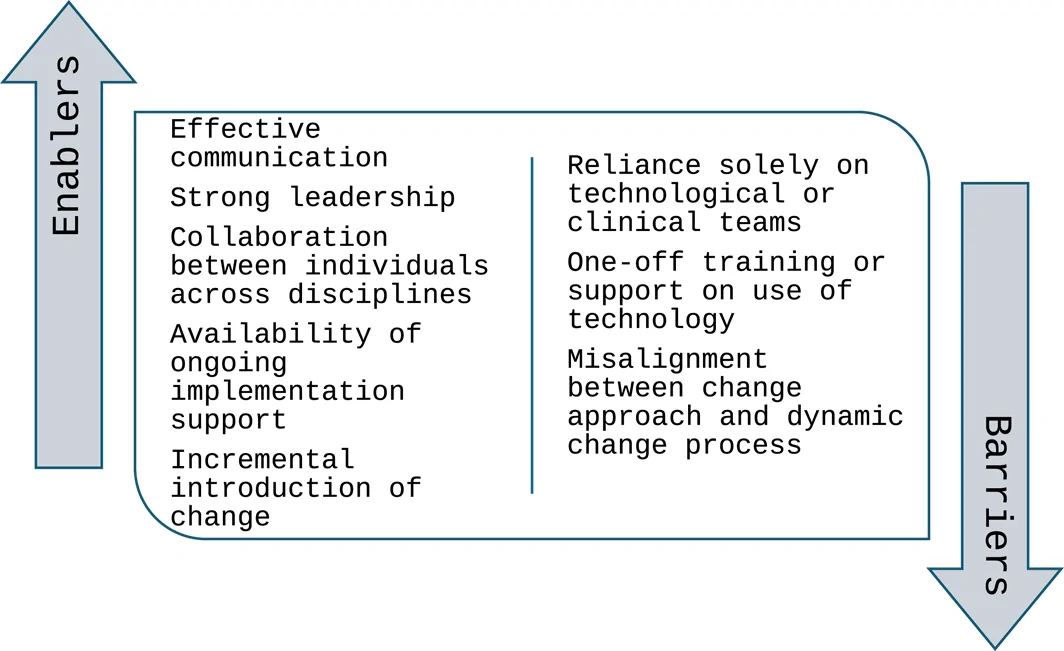
Diagrammatic representation of key enablers or barriers.

Commonly reported enablers underpinning successful application of change management models in healthcare settings included high quality communication among all parties involved and strong leadership [29, 33, 35]. Choosing a change management model that aligns with the values of the healthcare setting values and the importance of engagement from all stakeholders partaking in the project were also identified as central in improving a project's success [34, 37]. Figure 2 provides an overview of enablers or barriers identified across the included studies.

Specific enablers were however identified with relevance to changes associated with virtual or digitally enabled care. Success was considered more likely when individuals with technological, professional and local expertise and experience were able to collaborate in designing and delivering virtual and digital care changes with executive support [21, 30, 32]. Conversely, projects that solely rely on delivery by either technical or clinical teams may face risks in having limited engagement from all parties required to deliver and sustain the new approach to care provision. Approaches used to bring together the required multidisciplinary group included the establishment of multidisciplinary guiding teams that include representation from executives, those with technical, clinical and change management expertise to navigate the project lifecycle [21, 35, 36, 37, 40, 43]. Despite the reported value of such steering committees, the project‐based nature of such an approach and ability to sustain changes and respond to matters arising beyond the project lifecycle were not explored in the included studies.

Notable was the tendency for change projects involving new technology to focus on technology‐related changes by ensuring that individuals use the technology at the detriment of addressing the wider changes associated with introducing virtual and digitally enabled care. When focusing on the technology, one off training sessions were frequently used to upskill clinical teams [33]. In some cases, front‐line clinicians expressed dissatisfaction with this type of training approach because it contained too much information and was not sufficiently tailored, contributing to staff anxiety [21]. In the case of an EMR implementation, an attempt to address this challenge saw vendor training extended to place greater emphasis on simulation training. Rather than just learning how to use the system without recognition of the specific context, each ward established simulation labs that sought to enable clinicians to learn how to incorporate the digital systems into their workflows [21]. Findings suggest that whilst the tailored approach was valued, ongoing implementation support is critical for real‐life application of new technologies and the associated workplace changes that ensue to enable a smoother change process [21]. An example of ongoing implementation support as shown in a study introducing a medication barcoding system in which IT staff provided coaching to nursing staff in real‐time as the technology was introduced [30]. Findings suggest that sustaining change was enabled by the real‐time support to embed the new technology within the existing system and culture of care, reflected in other included studies [30, 36].

There was an apparent misalignment in the approach to using change management models and the requirement for dynamic change processes. For instance, application of Kotter's model was often described in a linear way that lacked recognition of the requirement for ongoing engagement and adjustment of the approach to engagement across the change process [43]. Integrating additional methods to monitor and respond to progress, such as using the PDSA method, may provide an enhanced approach [34].

Technology‐related changes were often addressed in the context of a wider overall change management approach in the context of large‐scale change, exemplified in an article reporting the introduction of an EMR system in an Australian hospital. Reflecting on the process undertaken, the Chief Information Officer noted the need to incrementally introduce and socialise the changes to be realised with frontline staff in a bid to balance seeking engagement with creating anxiety around disruption [21]. The incremental implementation of changes appeared to be influenced by whether staff were able to engage with a shared vision; some viewed the new system as a process of digitalisation of a paper system while others saw it as a process of digital transformation to leverage the EMR for innovation in the provision of care, with implications for benefits realization [21].

## 4 Discussion

### 4.1 Principal Findings

Through synthesis of 17 included articles, the review revealed that overarching strategic change models are commonly adopted in health service changes to support the introduction or integration of virtual care. Staged models such as Kotter's 8 Step Model are considered easy for both change managers and clinicians to engage with and follow. Despite the high proportion of included articles that reported the use of structured change methods, there was limited evidence about how the use of such models influenced the success of change management beyond providing a roadmap and process for change. It is apparent from the included articles that widely known change management models are often conceptualised and applied in a linear approach by healthcare change leaders and managers that does not account for the complexity of health systems function [44] or of the process of managing change [45].

Whilst change models provide a basis for planning and executing a change management process, case study reports in the included studies depict the management of virtual care change as contingent upon the selection of technology, the extent of stakeholder engagement throughout the project, the approach to training staff to engage with new technologies and workflows [21, 30, 37, 39]. The findings add to the current evidence base by consolidating knowledge of approaches used in virtual care change but also highlighted evidence gaps about how change sponsorship, methodologies, and implementation approaches collectively influence the likelihood of a successful change project.

Many of the included studies stated using a certain change methodology, but activities described did not always correspond to the steps, nor did they provide concrete examples of what was done, making it difficult to understand if the methodology was implemented as intended [41, 42]. Studies were often limited in their detail of whether and how change success was determined, what were the resources available to support the process of change management, and how this impacted the process.

### 4.2 Comparison With Prior Work

One approach often used in the included projects to address the dynamic nature of change management in integrating virtual care was to synthesise an overall strategic change approach with a dynamic process to capture and report change and improvement within a project, for example using continuous improvement methodology [34]. As a group of case studies, the included articles depicted a central contribution of leadership skills that were also drawn upon to select and combine change management methods relevant to the change issue in focus [35, 36]. Leadership skills implied in the detail of such case studies included understanding when and how to make use of frameworks, how to tailor and combine relevant to the change requirements and how to respond to the interpersonal and professional challenges that arise within the change management process. Such skills are reflected in the broader change management literature [22, 46]. The requirement for effective communication with all stakeholders was heightened in the context of virtual care change in which all stakeholders including vendors and technical staff need to be engaged and working towards a common outcome. Findings highlighted the perceived benefits of involving multidisciplinary teams early on encompassing all those who might be affected by the change.

In the context of virtual care change, there is a risk of a linear change process being adopted that is linked to the introduction of the technology without due consideration of the range of adaptations required of processes and people interacting with the technology [47]. Healthcare environments are complex, with multiple variables that interact in unpredictable ways in different contexts and for different patients. In the context of complex healthcare environments, change management must be adaptive and flexible [44, 45, 48]. Proactive approaches to plan for the implementation of change and for monitoring the change progress provide an avenue to navigate the health care environment and be responsive to issues that arise as virtual care is implemented. This was exemplified in extended literature by two studies that described the introduction of virtual care using integrated‐Promoting Action on Research Implementation in Health Services (i‐PARIHS) implementation framework [8, 49]. These two studies reported on the importance of planning for implementation and monitoring of the implementation for successful adoption of virtual care [8, 49].

### 4.3 Implications

Discrete activities such as opportunities for clinical staff to meet regularly with technical staff, providing targeted training, providing context specific resources, creating a one‐stop support for virtual care technologies and implementing pilots among others had been reported in virtual care implementation literature to assist with monitoring and implementation of virtual care technologies [49]. This review complements these activities in the context of managing a change management process associated with implementation of virtual care in three ways. Firstly, whilst useability assessments or simulations are often used and valued in virtual care implementation, providing ongoing, real‐time support through mentoring or coaching between clinical and technical staff is important. This may help staff to embed technical change and address technical challenges that arise over time and in context of their real‐life use of the technology. Secondly, recognising the variety of contexts in health service delivery, tailored training about use of virtual care processes and tools to needs of the team rather than relying on standardised technical training may be preferable for staff to understand how the technology and process may impact current practice and the wider implications of forthcoming change. For example, this approach may highlight a further set of challenges that need to be addressed as part of the change management. Finally, in the context of a high volume of change in healthcare settings and potential for change fatigue, a focus on incremental changes and gradual increases in the adoption of a new technology may enable users to understand and embrace virtual care change.

Tracking the implementation of a change management process is identified as best practice to achieve successful sustained change [50, 51]. Unlike approaches recommended in the change management literature [52], the included studies lacked evidence of methods that were used to track and determine the effectiveness or success of a change management process. Considering that most change initiatives do not proceed as planned, tracking and measuring change progresses is critical to ascertain adjustments required to keep change on track [52, 53]. A number of tools and resources are available in the wider management literature to monitor change progress that use milestones and metrics to assess and measure change performance, opportunities and outcomes [51, 52, 54]. Prosci Change Scorecard provides one such example in which the progress of the change could be measured across three performance level—individual, organisational and change management, to align change activities to the aim [55]. Considering range of change methodologies used, health organisations could develop a standard change management tracking tool that could be used across project. Future research could examine the validation and application of such tools to determine the success of change management methods in managing virtual care change. Wider evaluation can also inform implementation of such tools at health system level.

### 4.4 Limitations

The findings must be considered in light of the limitations of both the review process and the included studies. Whilst an expansive search was deployed in consultation with a medical librarian, the vast distribution of relevant articles across management, health and digital literature and use of differing terminologies across these disciplines means that some relevant articles may have been omitted. It is also possible that relevant information about change management in the context of virtual care is included in articles that did not focus primarily on the issue of change management. The included articles were case studies, which were generally limited in terms of their application to setting beyond the case study context. The case studies, while providing rich detail of change management methods used, were heterogenous in their reporting of information which limited the opportunity to draw conclusions about the impact of change management strategies across the body of work. Included studies also primarily focused on metropolitan contexts and the clinical and managerial perspectives of change management as opposed to having a consumer lens. Future research could examine grey literature as non‐peer reviewed articles were excluded from this study. This would assist with evaluation of change approaches that may not have been reported in published peer‐reviewed literature.

## 5 Conclusions

The growing use of virtual care as part of healthcare delivery is contributing to a high volume of change management that has a virtual care component. Technology must be considered within but not be the sole focus of change management that encompasses virtual care introduction or integration. Structured change management approaches can provide a useful framework for change processes but risk purporting a linear process that does not reflect the complexity of change in health service settings. Integrating structured change management approaches with dynamic tools that enable the capture of change management success is perceived as a useful strategy to support virtual care change.

## Funding

This work is funded by the National Health and Medical Research Council Partnership Scheme Grant No: 2015544.

## Conflicts of Interest

The authors declare no conflicts of interest.

## Supporting information


Supporting Information S1

Supporting Information S2

## Data Availability

Data sharing not applicable to this article as no datasets were generated or analysed during the current study.
