# Supplementary material for: Applying Change Models and Methods During a Period of Vast Digital Transformation: A Systematic Review of Practice in Healthcare
Source: Int J Health Plann Manage. 2026 May 21;41(5):1032–53. doi: 10.1002/hpm.70092 (PMC13551529; doi:10.1002/hpm.70092)
Supplement: Supplementary file 1 [file HPM-41-1032-s001.docx]

Supplementary file 2: Quality assessment of the included studies.

| **Quality appraisal questions** | **Arabi et al** | **Balsyman et al** | **Bird et al** | **Bousquet et al** | **Burns et al** | **Carman et al** | **Chang et al** | **Dyudina** | **Eden et al** | **Glegg et al** | **Krasuska et al** | **Lindsay et al** | **Ma et al** | **Pizzinni et al** | **Sale et al** | **Tomboccon et al** | **Tortorella et al** |
| --- | --- | --- | --- | --- | --- | --- | --- | --- | --- | --- | --- | --- | --- | --- | --- | --- | --- |
| Did the study address a clearly focused question / issue? | Yes | Yes | Yes | No | No | Yes | Yes | Yes | No | Yes | Yes | Yes | Yes | No | Yes | Yes | Yes |
| Is the research method (study design) appropriate for answering the research question? | Yes | Yes | Can't tell | Can't tell | Yes | Yes | Yes | Yes | Yes | Yes | Yes | Yes | Yes | Can't tell | No | Yes | Yes |
| Are both the setting and the subjects representative with regard to the population to which the findings will be referred? | Can't tell | Can't tell | Can't tell | Yes | Yes | No | Yes | Can't tell | Yes | Yes | No | No | Yes | No | Yes | Yes | Yes |
| Is the researcher’s perspective clearly described and taken into account? | Yes | No | Yes | Can't tell | Yes | Yes | Can't tell | Can't tell | No | Yes | Yes | Yes | No | No | Can't tell | No | No |
| Are the methods for collecting data clearly described? | Yes | No | No | Yes | Yes | Yes | Yes | No | No | Yes | Yes | No | Yes | No | Can't tell | No | Yes |
| Are the methods for analyzing the data likely to be valid and reliable? Are quality control measures used? | Can't tell | No | No | No | Yes | Can't tell | Yes | No | No | Yes | No | No | Yes | Can't tell | No | Can't tell | Can't tell |
| Was the analysis repeated by more than one researcher to ensure reliability? | Can't tell | Can't tell | Can't tell | Can't tell | Can't tell | Can't tell | Can't tell | No | Can't tell | Can't tell | Can't tell | Can't tell | No | No | Can't tell | No | No |
| Are the results credible, and if so, are they relevant for practice? | Can't tell | Can't tell | No | Yes | Yes | Can't tell | Yes | Yes | Yes | Yes | Yes | Can't tell | Yes | Yes | Can't tell | Yes | Yes |
| Are the conclusions drawn justified by the results? | Yes | Yes | Can't tell | Yes | Yes | No | Yes | Yes | Yes | Yes | Yes | Yes | Yes | Yes | Can't tell | Yes | Yes |
| Are the findings of the study transferable to other settings? | Can't tell | Yes | Can't tell | Yes | Yes | Can't tell | Yes | No | Yes | Yes | Yes | Yes | Can't tell | Can't tell | No | Can't tell | Can't tell |
| Adapted from Crombie, The Pocket Guide to Critical Appraisal; the critical appraisal approach used by the Oxford Centre for Evidence Medicine, checklists of the Dutch Cochrane Centre, BMJ editor’s checklists and the checklists of the EPPI Centre. | | | | | | | | | | | | | | | | | |
