# Supplementary material for: Applying Change Models and Methods During a Period of Vast Digital Transformation: A Systematic Review of Practice in Healthcare
Source: Int J Health Plann Manage. 2026 May 21;41(5):1032–53. doi: 10.1002/hpm.70092 (PMC13551529; doi:10.1002/hpm.70092)
Supplement: Supplementary file 2 [file HPM-41-1032-s002.docx]

| **Database/Journal** | **Search Strategy and Terms** |
| --- | --- |
| Medline Ovid | 1 Change Management/  2 (change management or change leadership or prosci or adkar or kotter or Lewin or re-aim or accelerated implementation).mp.  3 1 or 2  4 "Delivery of Health Care"/  5 exp Public Health/  6 exp Hospitals/ or Family Practice/  7 exp Primary Health Care/  8 exp Community Health Services/  9 exp Telemedicine/  10 (health care or healthcare or public health or hospital* or primary health care or family medicine or community health service* or general practice or telehealth or virtual care or virtual health or digital health).mp.  11 or/4-10  12 3 and 11  13 limit 12 to (English language and yr=”2019- current) |
| APA PsychInfo | 1 Change Management.mp.  2 (change management or change leadership or prosci or adkar or kotter or Lewin or re-aim or accelerated implementation).mp.  3 1 or 2  4 exp Public Health/  5 "Delivery of Health Care".mp.  6 exp Hospitals/  7 Family Practice.mp.  8 exp Primary Health Care/  9 Community Health Services.mp.  10 exp Telemedicine/  11 (health care or healthcare or public health or hospital* or primary health care or family medicine or community health service* or general practice or telehealth or virtual care or virtual health or digital health).mp.  12 or/4-11  13 3 and 12  14 limit 13 to (english language and yr="2019 -Current") |
| Business Source Premier | Set #1: ( (“change management” or “change leadership” or prosci or adkar or kotter or Lewin or re-aim or “accelerated implementation”) ) AND ( (“health care” or healthcare or “public health” or hospital* or “primary health care” or “family medicine” or “community health service*” or “general practice” or telehealth or “virtual care” or “virtual health” or “digital health”) )  Set #2: ( (“change management” or “change leadership” or prosci or adkar or kotter or Lewin or re-aim or “accelerated implementation”) ) AND ( (“health care” or healthcare or “public health” or hospital* or “primary health care” or “family medicine” or “community health service*” or “general practice” or telehealth or “virtual care” or “virtual health” or “digital health”) )  Set #3: ( (“change management” or “change leadership” or prosci or adkar or kotter or Lewin or re-aim or “accelerated implementation”) ) AND ( (“health care” or healthcare or “public health” or hospital* or “primary health care” or “family medicine” or “community health service*” or “general practice” or telehealth or “virtual care” or “virtual health” or “digital health”) )  Filter Years to 2019 – 2023 and tick option for English language only |
| Scopus | ( TITLE-ABS-KEY ( ( "change management" OR "change leadership" OR prosci OR adkar OR kotter OR lewin OR re-aim OR "accelerated implementation" ) ) AND TITLE-ABS-KEY ( ( "health care" OR healthcare OR "public health" OR hospital* OR "primary health care" OR "family medicine" OR "community health service*" OR "general practice" OR telehealth OR "virtual care" OR "virtual health" OR "digital health" ) ) ) AND ( LIMIT-TO ( PUBYEAR , 2023 ) OR LIMIT-TO ( PUBYEAR , 2022 ) OR LIMIT-TO ( PUBYEAR , 2021 ) OR LIMIT-TO ( PUBYEAR , 2020 ) OR LIMIT-TO ( PUBYEAR , 2019 ) ) AND ( LIMIT-TO ( LANGUAGE , "English" ) ) |
